# Supplementary material for: Adsorption Characteristics of Gas Molecules Adsorbed on Graphene Doped with Mn: A First Principle Study
Source: Molecules. 2022 Apr 2;27(7):2315. doi: 10.3390/molecules27072315 (PMC9000528; doi:10.3390/molecules27072315)
Supplement: Supplementary file 1 [file molecules-27-02315-s001.zip › molecules-1644008-supplementary.pdf]

molecules

Supplementary Materials

Adsorption Characteristics of Gas Molecules Adsorbed on Graphene Doped with Mn: A First Principle Study

Tingyue Xie [1,2], Ping Wang [2], Cuifeng Tian [2], Guozheng Zhao [1], Jianfeng Jia [1], Chaozheng He [3], Chenxu Zhao [3,*] and Haishun Wu [1,*]

1 Key Laboratory of Magnetic Molecules and Magnetic Information Materials of Ministry of Education, School of Chemistry, Materials Science of Shanxi Normal University, Taiyuan 030006, China; tingyuexie@126.com (T.X.); zhaoguozheng@sxnu.edu.cn (G.Z.); jiajf@dns.sxnu.edu.cn (J.J.)
2 School of Physical and Electronics Science, Shanxi Datong University, Datong 037009, China; wangping061226@aliyun.com (P.W.); cftian_050@sxdtdx.edu.cn (C.T.)
3 Institute of Environmental and Energy Catalysis, School of Materials Science and Chemical Engineering, Xi'an Technological University, Xi'an 710021, China; hecz2019@xatu.edu.cn
* Correspondence: zhaochenxu@xatu.edu.cn (C.Z.); wuhs@sxnu.edu.cn (H.W.)

Table S1. The absorption height (*h*, Å) of Mn atoms; Average distance between the dopant and its neighboring C or N atoms (*d*, Å); Amount of charge transfer (Δq, e, the acquisition or loss of electrons is indicated by the "+" or "-") from Mn atom to supports; Binding energies (E_b, eV); The total magnetic moments (M, μ_B).

Configuration	*h*	*d*	Δq	E_b	*M*
MnSV-GN	1.40	1.83	-0.88	-6.12	3.00
MnDV-GN	0.70	1.99	-1.06	-5.86	2.94
MnN_4-GN	0.03	1.92	-1.28	-6.61	3.05

Table S2. The uplift height of Mn atoms; The -IpCOHP values of various atomic interactions; The adsorption energies (E_{ad}) of gas molecules on MnSV-GN, MnDV-GN and MnN_4-GN; Total -IpCOHP values of Mn-C and Mn-O for CH_2O; -IpCOHP values of Mn-S for H_2S; -IpCOHP values of Mn-N for HCN.

Configurations	Properties	Gas Molecules		
		CH_2O	H_2S	HCN
**MnSV-GN**	-IpCOHP(eV)	4.86	2.55	3.02
	Δh(Å)	0.04	0.02	0.03
	E_{ad}(eV)	-1.86	-0.83	-1.11
**MnDV-GN**	-IpCOHP(eV)	4.41	1.68	2.43
	Δh(Å)	0.14	0.07	0.11
	E_{ad}(eV)	-1.07	-0.71	-0.89
**MnN_4-GN**	-IpCOHP(eV)	3.44	0.66	1.21
	Δh(Å)	0.60	0.15	0.31
	E_{ad}(eV)	-0.44	-0.32	-0.35

Table S3. The charge changes of adsorbed gases (Δq); Total magnetic moment (M) after gas adsorption; Change of magnetic moment (ΔM) before and after gas adsorption (Δq, the acquisition or loss of electrons is indicated by the "+" or "-").

Gas	CH_2O			H_2S			HCN		
**Configuration**	**Δq(e)**	**M(μ_B)**	**ΔM(μ_B)**	**Δq(e)**	**M(μ_B)**	**ΔM(μ_B)**	**Δq(e)**	**M(μ_B)**	**ΔM(μ_B)**
MnSV-GN	0.41	1.00	2.00	-0.01	1.00	2.00	0.18	1.00	2.00
MnDV-GN	0.40	1.94	1.00	-0.07	2.84	0.10	0.09	2.84	0.10
MnN_4-GN	0.42	2.90	0.15	-0.06	3.05	0.00	0.05	3.01	0.04

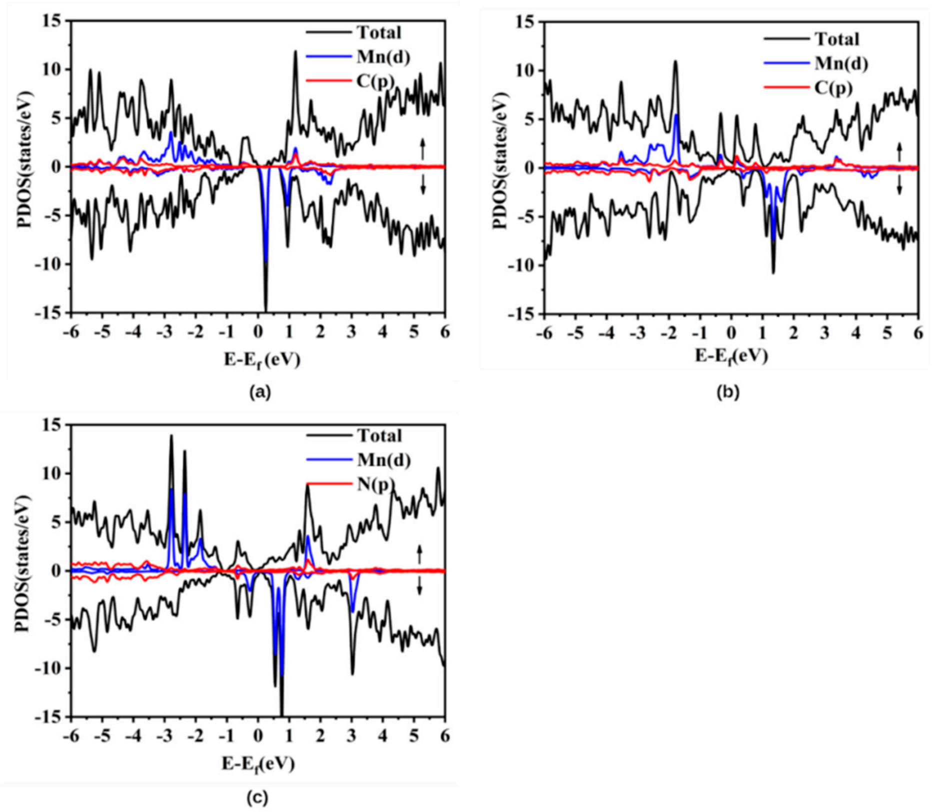

Figure S1. Projected density of states (PDOS) of (**a**) MnSV-GN, (**b**) MnDV-GN and (**c**) MnN_4-GN. The arrow up and arrow down indicate the spin up and spin down states, respectively.

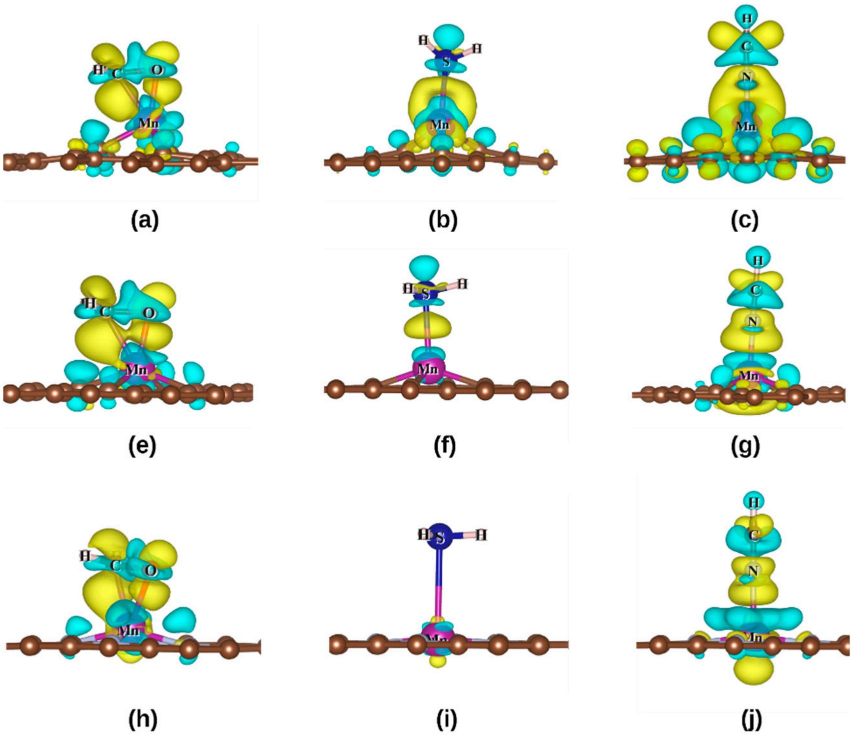

Figure S2. Charge density difference ($\Delta\rho = \rho_{A+B} - \rho_A - \rho_B$) of (**a**) CH_2O/MnSV-GN, (**b**) H_2S/MnSV-GN, (**c**) HCN/MnSV-GN, (**e**) CH_2O/MnDV-GN, (**f**) H_2S/MnDV-GN, (**g**) HCN/MnDV-GN, (**h**) CH_2O/MnN_4-GN, (**i**) H_2S/ MnN_4-GN and (**j**) HCN/ MnN_4-GN. The accumulation and depletion of electrons are represented by the yellow and cyan regions, respectively, isosurface value: 0.003e Å^{-3}.

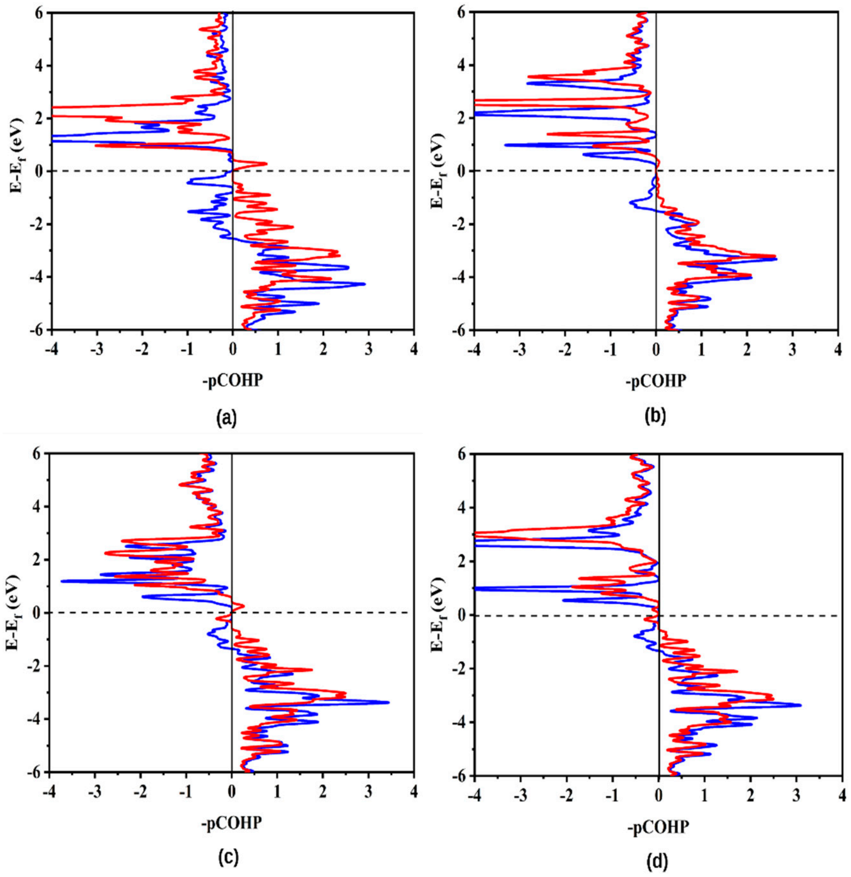

Figure S3. -pCOHP curves for the dopant Mn and its neighboring C interactions (**a**) MnSV-GN (**b**) CH_2O/MnSV-GN, (**c**) H_2S/MnSV-GN and (**d**) HCN/MnSV-GN in spin-polarized (magnetic) calculations. α-spin (spin up) and β-spin (spin down) is indicated by blue color and red color.

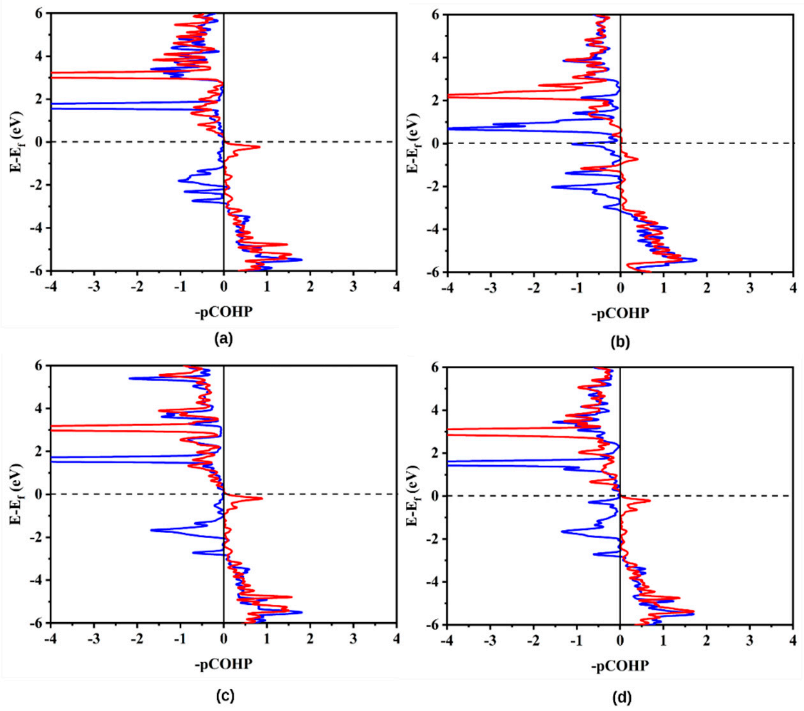

Figure S4. -pCOHP curves for the dopant Mn and its neighboring N interactions (**a**) MnN_4-GN (**b**) CH_2O/MnN_4-GN, (**c**) H_2S/MnN_4-GN and (**d**) HCN/MnN_4-GN in magnetic (spin-polarized) calculations. α-spin in blue color and β-spin in red color in the magnetic calculations.

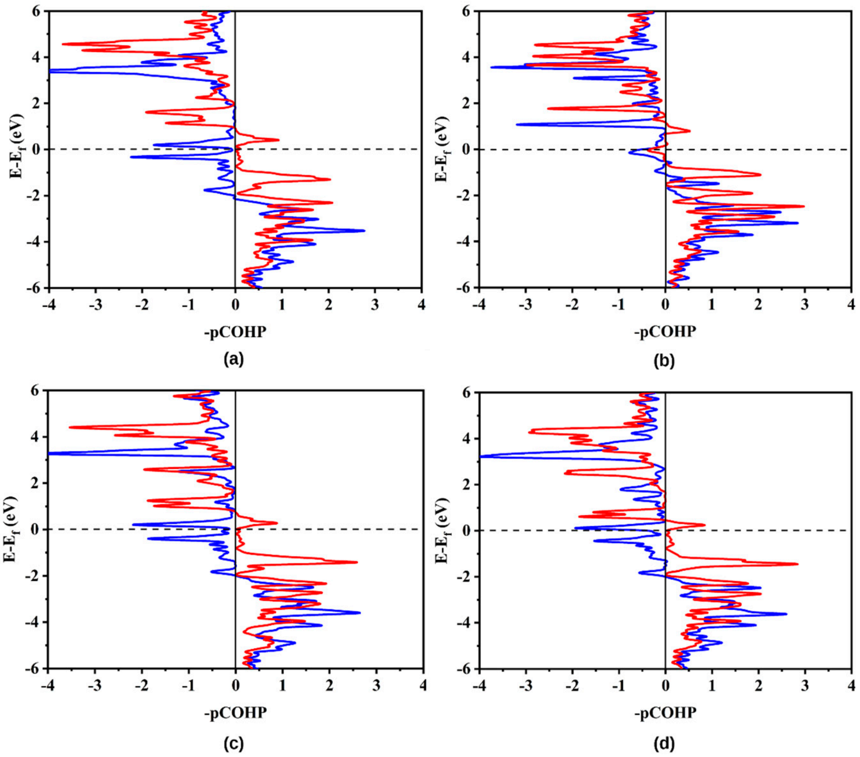

Figure S5. -pCOHP curves for the dopant Mn and its neighboring C interactions (**a**) MnDV-GN (**b**) CH_2O/MnDV-GN, (**c**) H_2S/MnDV-GN and (**d**) HCN/MnDV-GN in magnetic (spin-polarized) calculations. α-spin in blue color and β-spin in red color in the magnetic calculations.

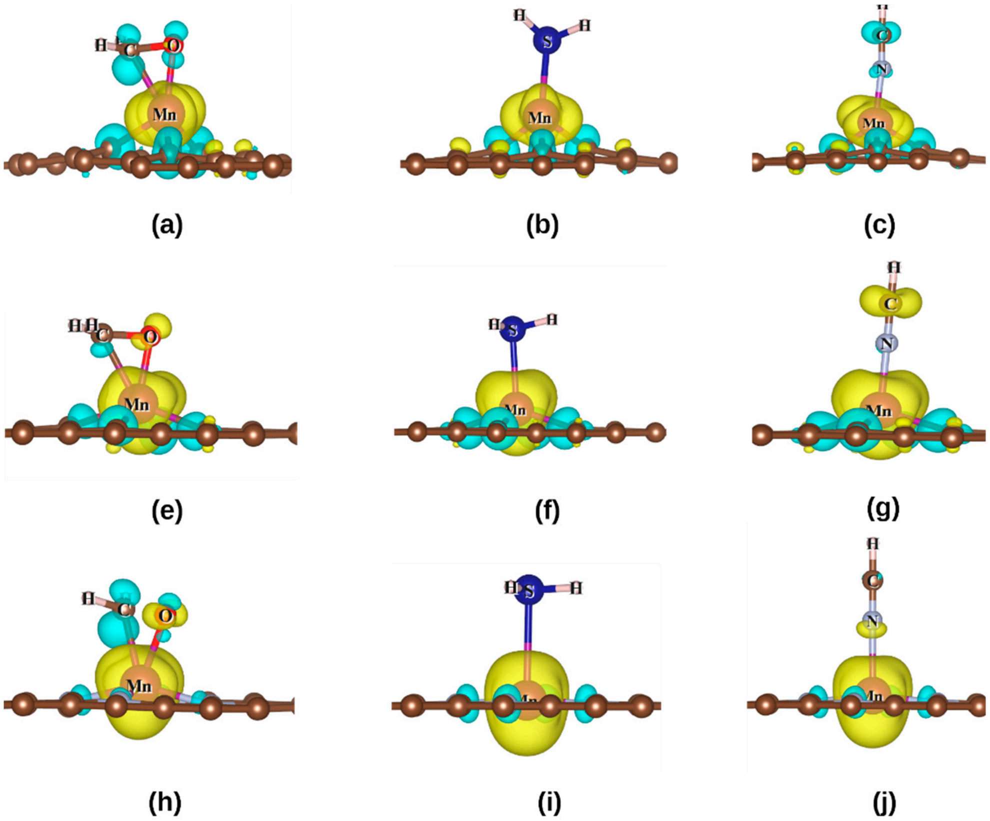

Figure S6. The spin density of (**a**) CH_2O/MnSV-GN, (**b**) H_2S/MnSV-GN, (**c**) HCN/MnSV-GN, (**e**) CH_2O/MnDV-GN, (**f**) H_2S/MnDV-GN (**g**) HCN/MnDV-GN, (**h**) CH_2O/MnN_4-GN, (**i**) H_2S/ MnN_4-GN and (**j**) HCN/ MnN_4-GN. (spin up (yellow) and spin down (cyan) isosurface value: 0.005e Å^{-3}).
